# Supplementary figures and images for: High allelic diversity in Arabidopsis NLRs is associated with distinct genomic features
Source: EMBO Rep. 2024 Mar 25;25(5):14. doi: 10.1038/s44319-024-00122-9 (PMC11093987; doi:10.1038/s44319-024-00122-9)

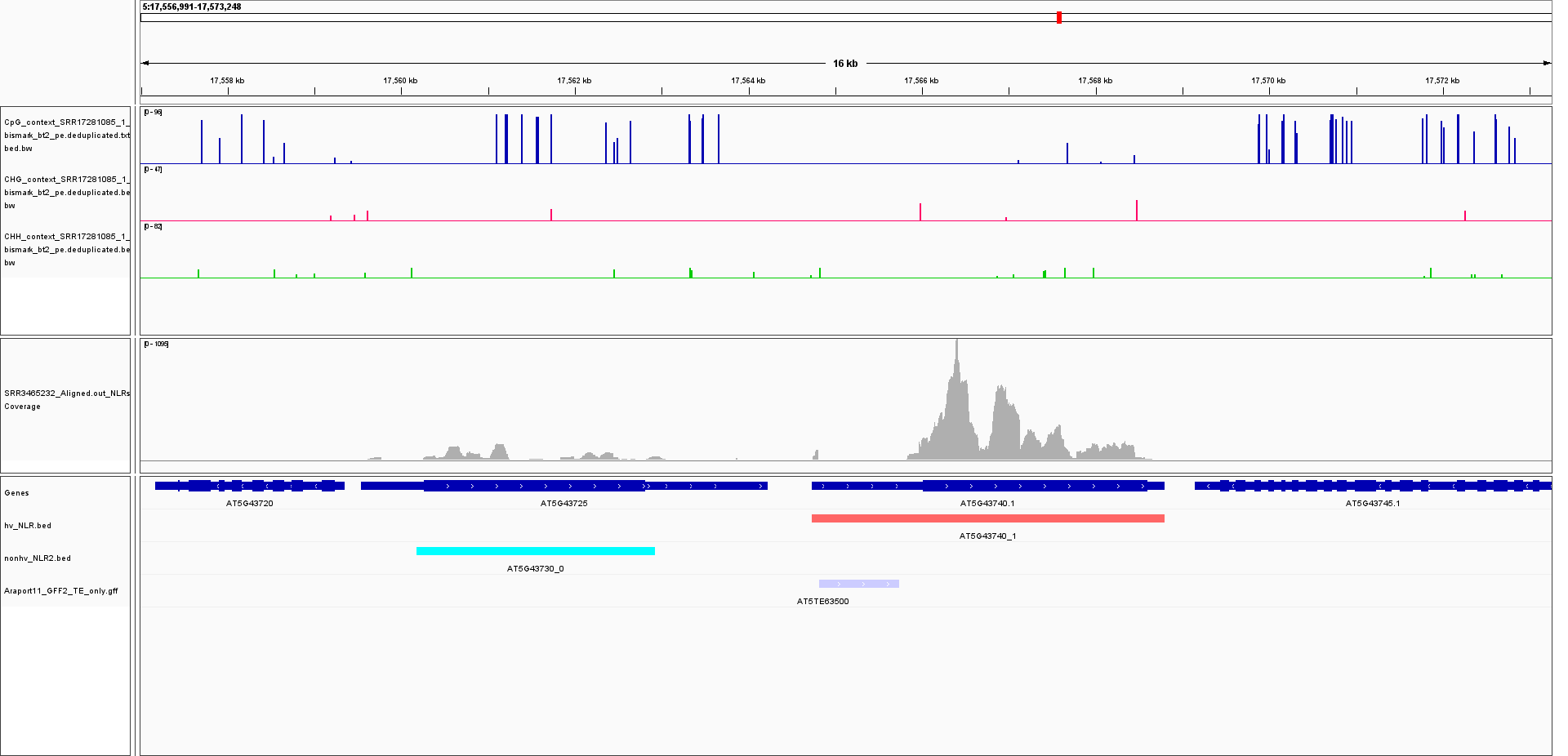

Supplement: Supplementary file 7 — Source data Fig. 7 [file 44319_2024_122_MOESM7_ESM.zip › Figure 7/7A/uncropped_igv_screenshot.png]

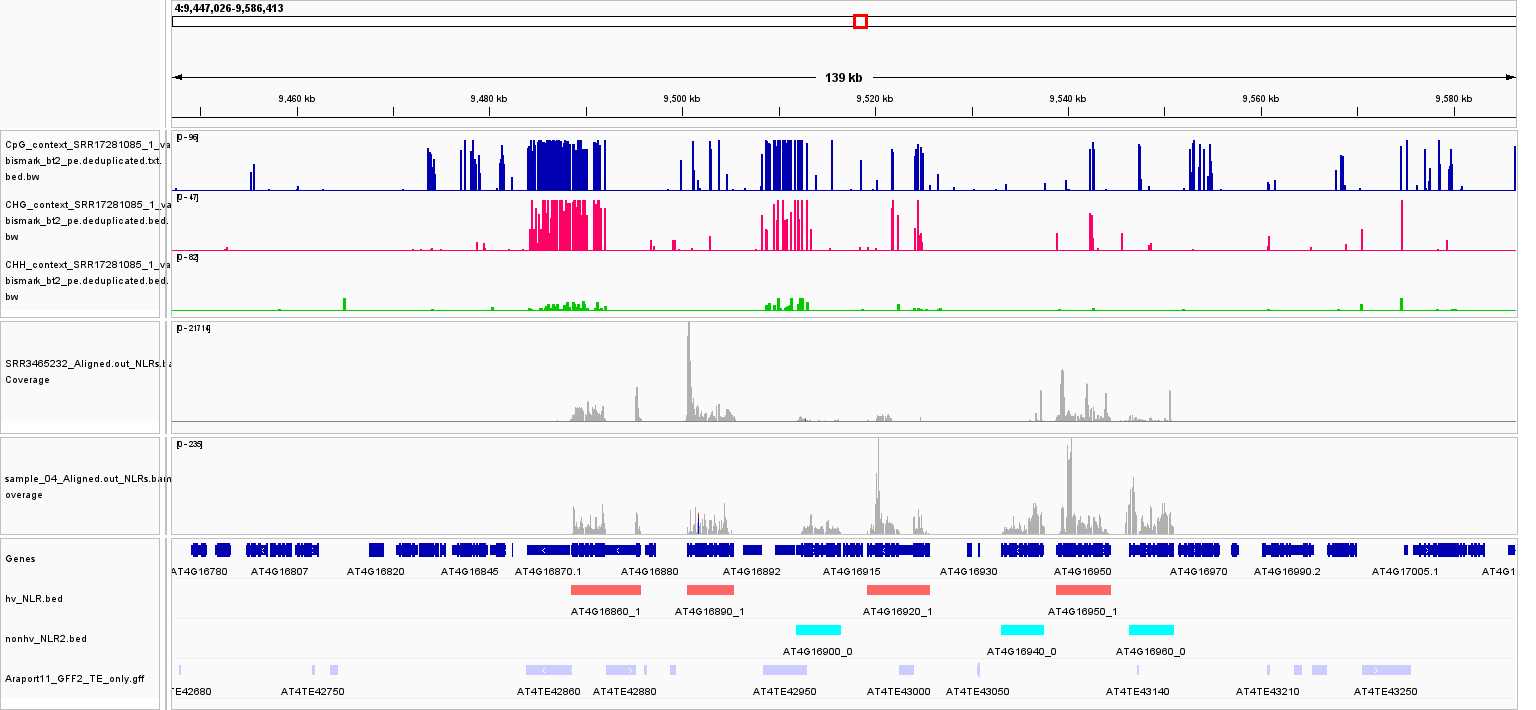

Supplement: Supplementary file 8 — Figure EV Source Data [file 44319_2024_122_MOESM8_ESM.zip › EV Figure 2/EV 2AB/rpp4_uncropped_igv_screenshot.png]

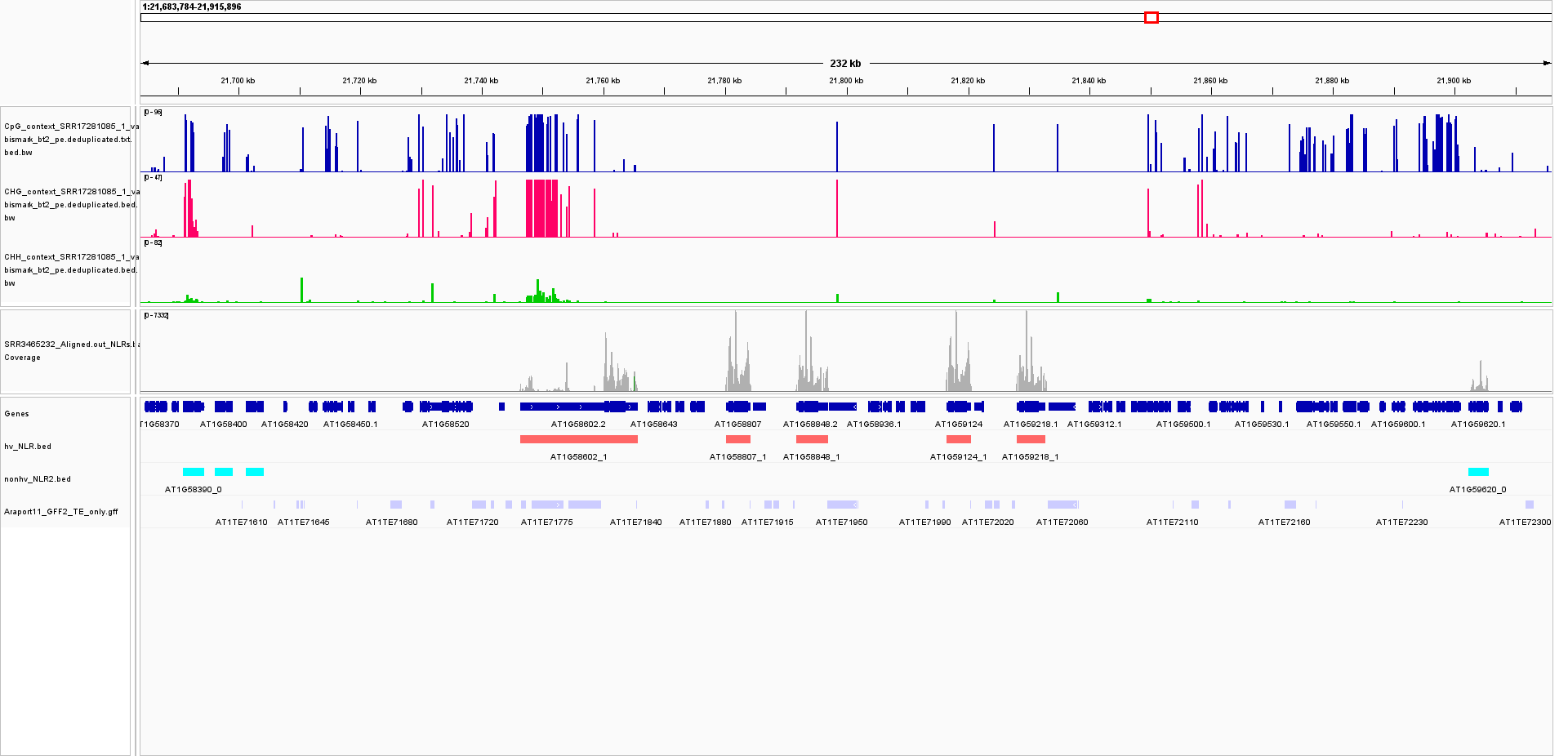

Supplement: Supplementary file 8 — Figure EV Source Data [file 44319_2024_122_MOESM8_ESM.zip › EV Figure 2/EV 2AB/rpp7_uncropped igv screenshot.png]
