# Supplementary material for: High allelic diversity in Arabidopsis NLRs is associated with distinct genomic features
Source: EMBO Rep. 2024 Mar 25;25(5):14. doi: 10.1038/s44319-024-00122-9 (PMC11093987; doi:10.1038/s44319-024-00122-9)
Supplement: Supplementary file 10 — Expanded View Figures [file 44319_2024_122_MOESM10_ESM.pdf]

Expanded View Figures

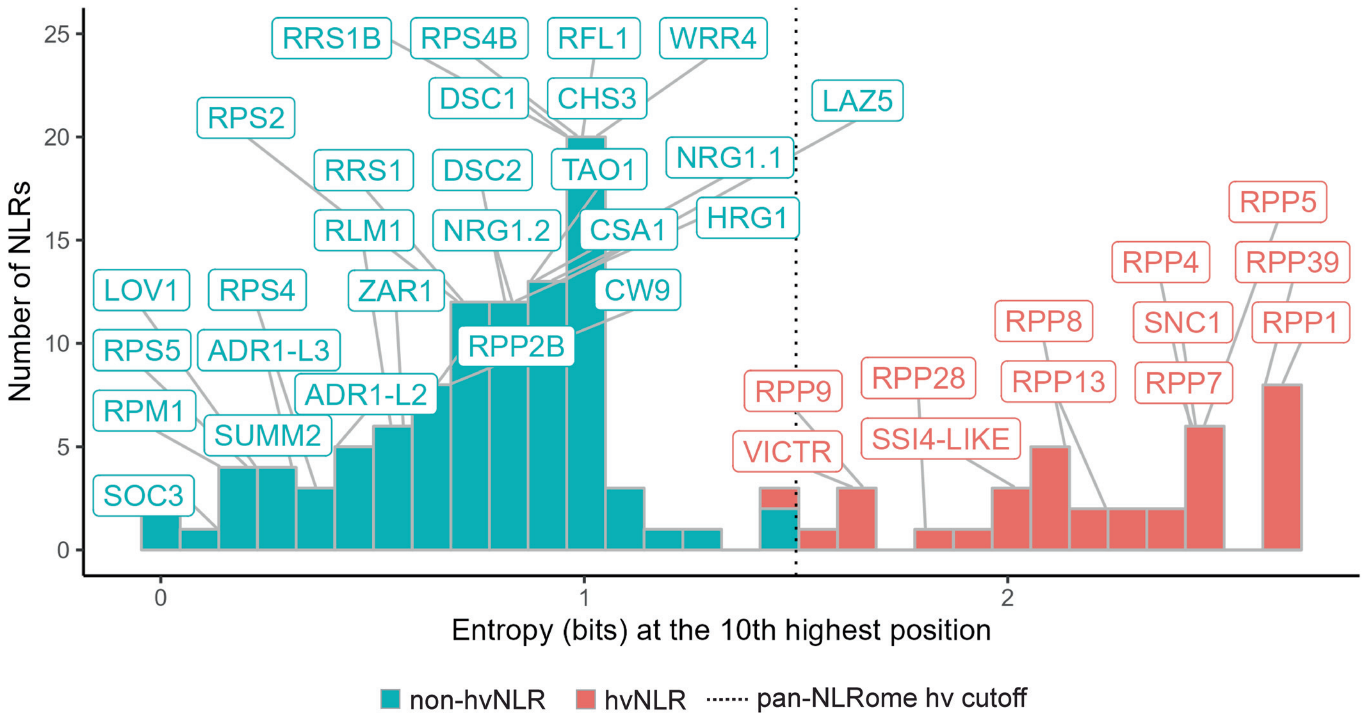

Figure EV1. ***Arabidopsis*** **Col-0 Shannon entropy at the tenth highest amino acid position.**

Distribution of NLR Shannon entropy per sequence at the tenth highest amino acid position as shown by a histogram with 30 bins. Named NLRs with previous functional characterization are labeled on the graph. The designation of hvNLR is entropy of >1.5 bits at the tenth highest position across the *Arabidopsis* NLRome, as shown by the dashed line. Source data are available online for this figure.

 © The Author(s)

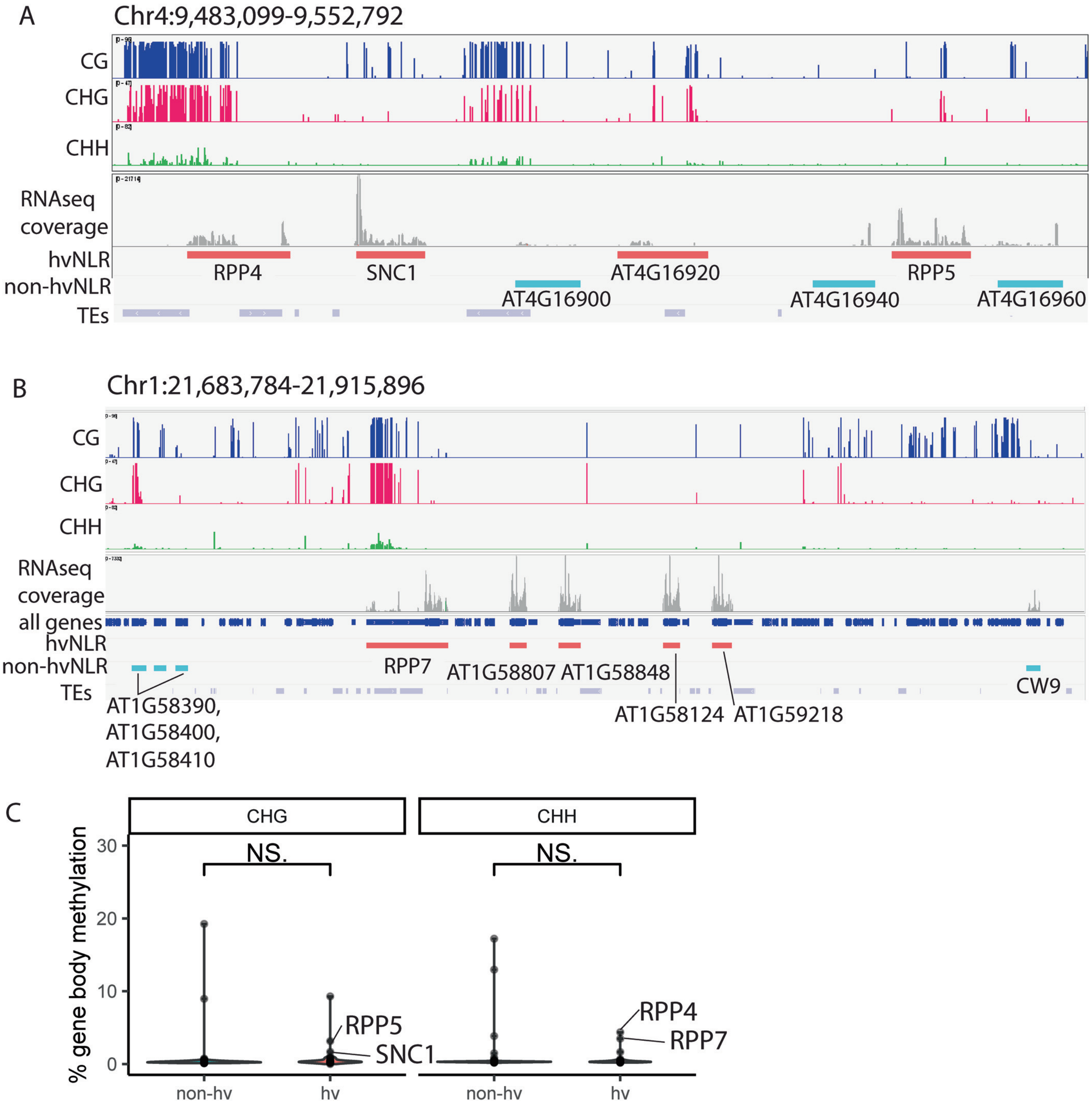

Figure EV2. Multi-context methylation of two NLR clusters likely due to recent TE insertion.

(**A**, **B**) Integrative Genomics Viewer screenshot of methylation, RNAseq coverage, and TE proximity of the *RPP4* and *RPP7* clusters in rosette leaf tissue. (**C**) % CHG and CHH gene body methylation of non-hv and hvNLRs in rosette leaf tissue. Named *RPP4* and *RPP7* cluster members are labeled. Data Information: (**C**) for both comparisons, $n = 97$ non-hvNLRs and $n = 35$ hvNLRs, with n referring to the number of genes tested. Significance shown is the result of unpaired Wilcoxon rank-sum tests with Benjamini–Hochberg correction for multiple testing. "n.s." indicates a P value > 0.05. Source data are available online for this figure.

© The Author(s)

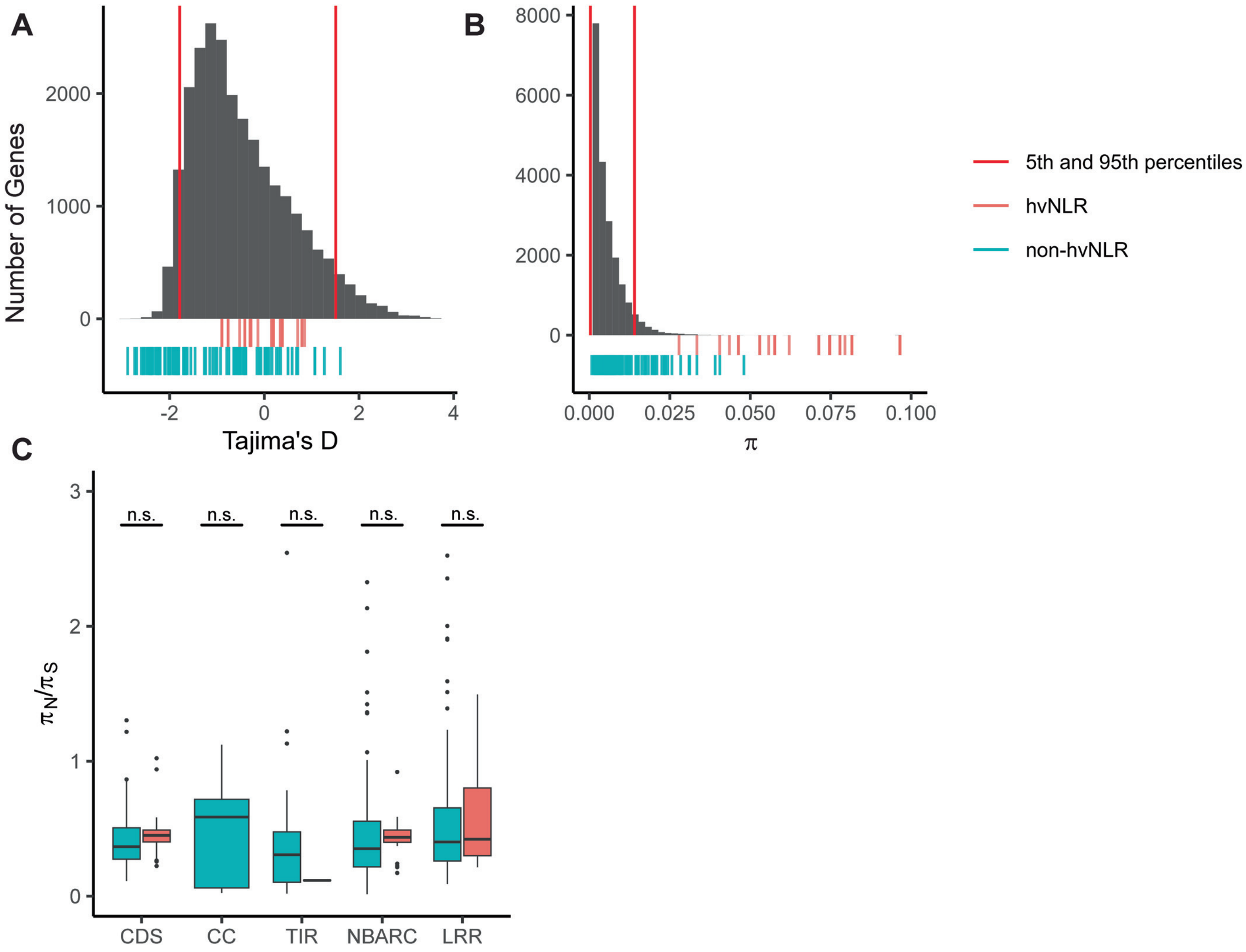

Figure EV3. Comparison of NLR population genetics statistics to the empirical distribution; per NLR domain π_N/π_S.

(**A**, **B**) Empirical distribution of Tajima's D and π calculated on coding sequences of *Arabidopsis* shown as a histogram with 50 bins. The position of hv and non-hvNLRs shown via rug plot beneath the histogram, as well as the 5th and 95th percentiles of the distribution. (**C**) π_N/π_S calculated per domain. Data Information: (**C**) horizontal black lines denote median values within each box; boxes range from the 25th to 75th percentile of each group's distribution of values; whiskers extend no further than 1.5× the interquantile range of the hinge. Data beyond the end of the whiskers are outlying points and are plotted individually. Significance shown is the result of an unpaired Wilcoxon rank-sum test with Benjamini–Hochberg correction for multiple testing. "n.s." indicates a *P* value > 0.05. Source data are available online for this figure.

 © The Author(s)

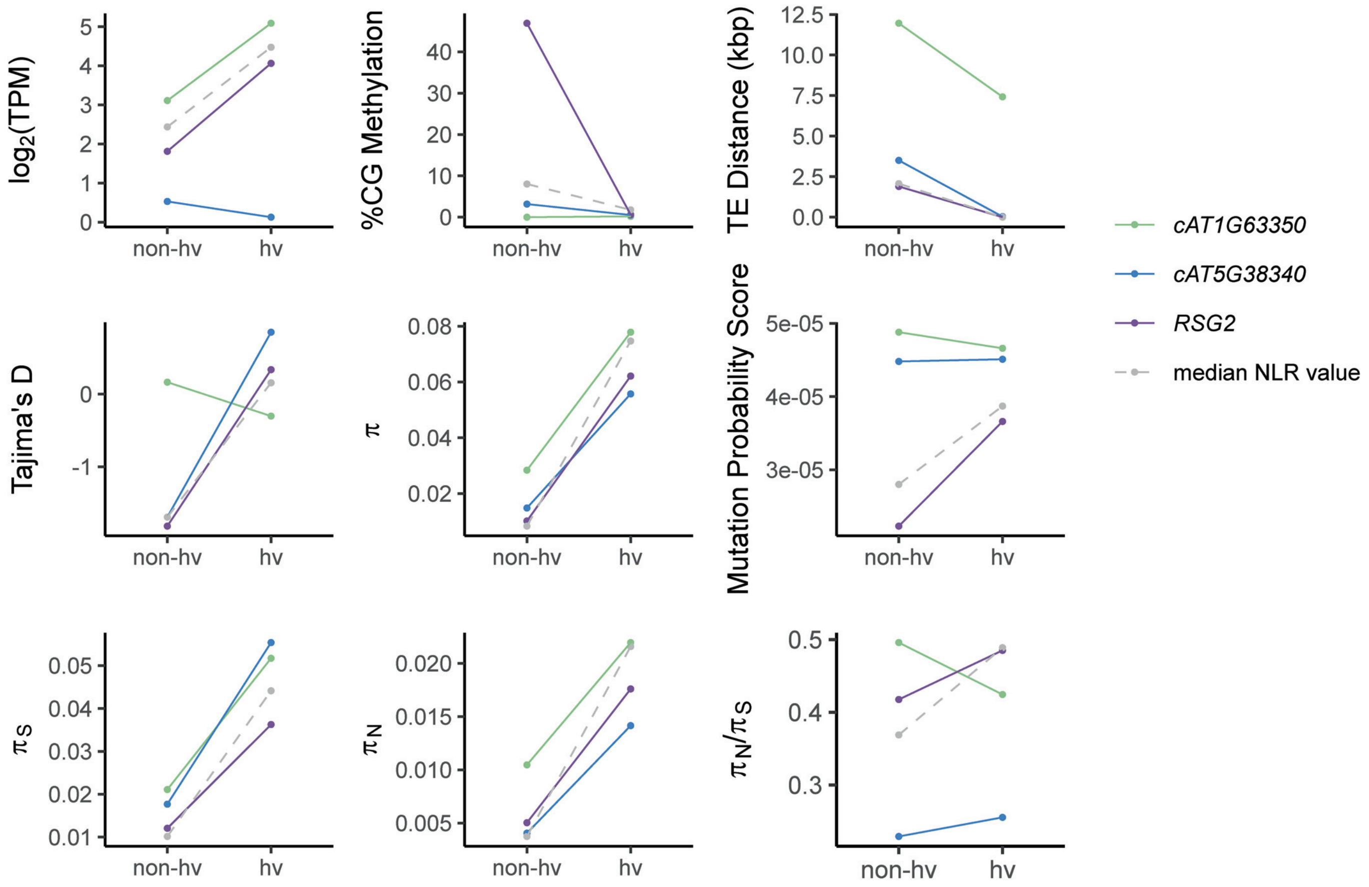

Figure EV4. Intra-cluster comparison of neighboring hv and non-hvNLRs.

Described genomic features and population genetics statistics are shown for hv and non-hvNLR pairs in three clusters: *RSG2*, *cAT1G63350*, and *cAT5G38340*. The median of all hv and non-hvNLR values is shown by a grey dotted line. Expression (log_2 (transcripts per million (TPM))) and % CG methylation shown is from rosette leaf tissue. Source data are available online for this figure.
